# Surgical Hyperspectral imaging and Indocyanine green Near-infrared Examination (SHINE) for brain arteriovenous malformation resection: a case report on how to visualize perfusion

**DOI:** 10.3389/fsurg.2024.1477920

**Published:** 2024-10-18

**Authors:** Johannes Wach, Ferdinand Weber, Martin Vychopen, Felix Arlt, Annekatrin Pfahl, Hannes Köhler, Andreas Melzer, Erdem Güresir

**Affiliations:** ^1^ Department of Neurosurgery, University Hospital Leipzig, Leipzig, Germany; ^2^ Innovation Center Computer Assisted Surgery, Faculty of Medicine, Leipzig University, Leipzig, Germany

**Keywords:** brain AVM, case report, hyperspectral imaging, intraoperative imaging, indocyanine green, image-guided surgery

## Abstract

**Background and importance:**

Arteriovenous malformations (AVMs) are complex vascular anomalies that pose significant risks, including intracranial hemorrhage and neurological deficits. Surgical resection is the preferred treatment, requiring precise intraoperative imaging to ensure complete removal while preserving critical structures. This case report presents the first combined use of hyperspectral imaging (HSI) and indocyanine green video angiography (ICG VA) to visualize perfusion during brain AVM surgery, highlighting the potential benefits of these advanced imaging techniques.

**Case description:**

A 66-year-old male presented with chronic headaches but no neurological deficits. MRI revealed a superficial AVM in the left frontal lobe within the superior frontal sulcus, measuring approximately 2.4 cm. The AVM was fed by feeders from the pericallosal artery, callosomarginal artery, and middle cerebral artery (MCA) branches, with drainage through a dilated cortical vein into the superior sagittal sinus. Preoperative embolization of two MCA feeding branches was performed, followed by microsurgical resection with ICG VA and HSI.

**Conclusions:**

This case report demonstrates the successful application of HSI and ICG VA in brain AVM surgery. The combined use of these technologies provided comprehensive intraoperative assessment, enhancing surgical precision and safety. The integration of HSI offers non-invasive, contrast-agent-free imaging, potentially improving outcomes by enabling detailed perfusion mapping. Future studies should explore the broader applications of these imaging modalities in neurovascular practice.

## Graphical Abstract

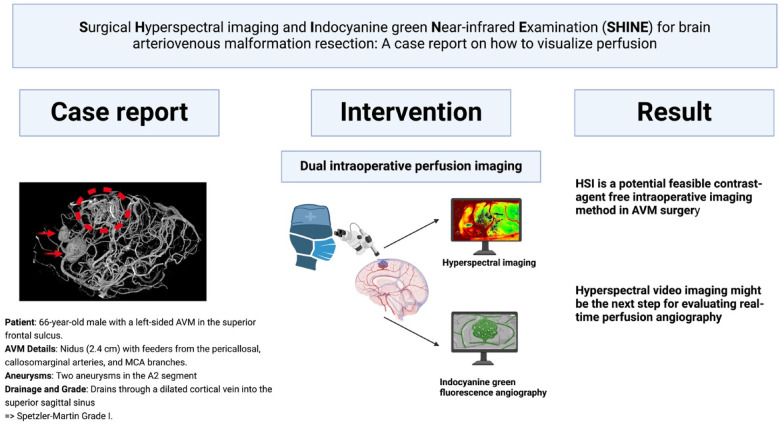

## 1 Introduction

Arteriovenous malformations (AVMs) are intricate vascular anomalies where a tangled web of arteries and veins bypass the capillary system (1). This abnormal connection poses significant risks, including intracranial hemorrhage, seizures, and progressive neurological deficits (2). Surgical resection is often the treatment of choice for AVMs, necessitating precise intraoperative imaging to ensure complete removal while preserving critical vascular structures in unruptured Spetzler-Martin grade I and II patients (3). A fundamental principle of neurosurgical AVM treatment is to maintain perfusion by avoiding unnecessary vascular compromise. In the resection of intracerebral AVMs, this involves excluding uninvolved vessels and protecting nidal draining veins until arterial feeders are disconnected (1). Indocyanine green (ICG), a fluorescein-like dye, can be intravenously injected to help delineate vascular anatomy under direct visualization. ICG videoangiography (VA) is particularly useful as it allows the surgeon to directly and immediately assess the integrity of vessels under the intraoperative microscope (4, 5). ICG VA has become a valuable tool in neurosurgery, particularly for AVM management (6). ICG is a fluorescent dye that, when excited by near-infrared light, provides real-time visualization of blood flow (4, 5). This allows surgeons to delineate vascular structures clearly and verify complete resection of AVMs while protecting vital adjacent vessels (6). The technique was notably integrated into surgical microscopes by Raabe et al. in 2003, significantly enhancing intraoperative assessment during vascular neurosurgery (4). ICG VA is praised for its simplicity, rapid deployment, and real-time feedback. Despite the low toxicity profile (e.g., hypersensitivity reactions in iodine allergy) of ICG VA, modern intraoperative imaging methods strive to work without the application of contrast agents (7, 8). Hyperspectral imaging (HSI) is a novel imaging modality that captures a wide spectrum of light beyond the visible range (9). This technology provides detailed spectral information, enabling the differentiation of various tissue types based on their unique spectral signatures. Unlike ICG, HSI does not require the application of any contrast agents, making it a non-invasive alternative with minimal patient risk. HSI offers fast image acquisition and can be integrated into the surgical workflow without significantly prolonging operation times (10). Recent advancements have showcased HSI's potential in neurosurgery, particularly in tumor resection and tissue differentiation (9, 10). However, the present case illustrates the first use of intraoperative HSI in brain AVM surgery ability to demonstrate vascular structures and tissue characteristics, which is crucial for the accurate resection of AVMs.

## 2 Case description

### 2.1 Baseline case characteristics

**Figure 1:**
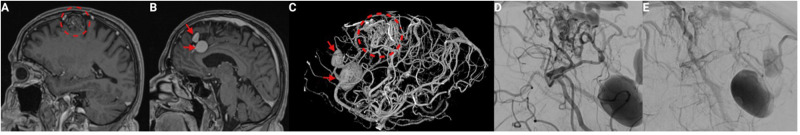
Preoperative imaging of a superficial arteriovenous malformation (AVM) with flow-induced aneurysms. **(A)** Sagittal T1-weighted Gd-enhanced MRI demonstrating the superficial Martin-Spetzler grade I AVM located in the left frontal lobe within the superior frontal sulcus (dashed red circle). **(B)** Sagittal T1-weighted Gd-enhanced MRI highlighting two large flow-related aneurysms on the feeding arteries: one on the pericallosal artery and the other on the callosomarginal artery (red arrows). **(C)** 3D reconstructed angiogram showing the AVM with its complex vascular architecture and flow-induced aneurysms (red arrows indicate aneurysms, dashed red circle indicates the AVM nidus). (**D)** Angiogram showing the AVM prior to embolization, highlighting the feeding arteries. **(E)** Angiogram after embolization of two feeding branches from the middle cerebral artery, showing reduced blood flow to the AVM.

The 66 years old male patient underwent magnet resonance imaging (MRI) because of chronic headaches. Clinical examination revealed no neurological deficits. MRI presented a superficial AVM located in the left frontal lobe, within the superior frontal sulcus (see Figures 1A,B). Further diagnostic workflow was performed with digital substraction angiography (DSA) (see Figures 1C,D). The AVM had a nidus size of approximately 2.4 cm. It received feeders from the pericallosal artery, the callosomarginal artery, and a branch of the middle cerebral artery (MCA) with drainage through a dilated cortical vein into the superior sagittal sinus. Imaging also revealed flow-related aneurysms in the A2 segment from the pericallosal and the callosomarginal artery sized 20, and 12 mm, respectively. Finally, the AVM was classified as Spetzler-Martin Grade I.

### 2.2 HSI measurements

The TIVITA® Tissue HSI camera system (Diaspective Vision, Germany) is a CE-certified, mobile hyperspectral imaging device designed for clinical use. It enables non-invasive, contrast-agent free intraoperative image capture with both qualitative and quantitative analysis (11–13). This system assesses four key tissue parameters: tissue oxygenation (StO_2_), near-infrared perfusion index (NPI), tissue hemoglobin index (THI), and tissue water index (TWI). It operates by analyzing the light reflection and absorption of substances like oxy-/deoxyhemoglobin and water, providing spatial information on tissue oxygenation, perfusion, and water content (14). HSI measurements with the TIVITA® system evaluate four parameters across the following specific wavelength ranges (11):

• StO_2_: Tissue oxygenation (1-mm depth; 500–650 nm and 700–815 nm)
• NPI: Near-infrared perfusion index (4–6-mm depth; 655–735 nm and 825–925 nm)
• THI: Tissue hemoglobin index (530–590 nm and 785–825 nm)
• TWI: Tissue water index (880–900 nm and 955–980 nm)

StO_2_ is expressed in percentage, while NPI, THI, and TWI are shown as index values (0–100). These parameters are presented as color-coded images, with red/yellow indicating high values (50–100) and green/blue representing low values (0–50). The distance between the investigated brain tissue and the camera is standardized at 50 cm. The camera is integrated in a mobile cart with an attached computer performing the chemical color imaging procedure. The acquisition time of a single intraoperative hyperspectral image is 10 s (see Supplementary Figure) (15).

### 2.3 Perioperative course

**Figure 2:**
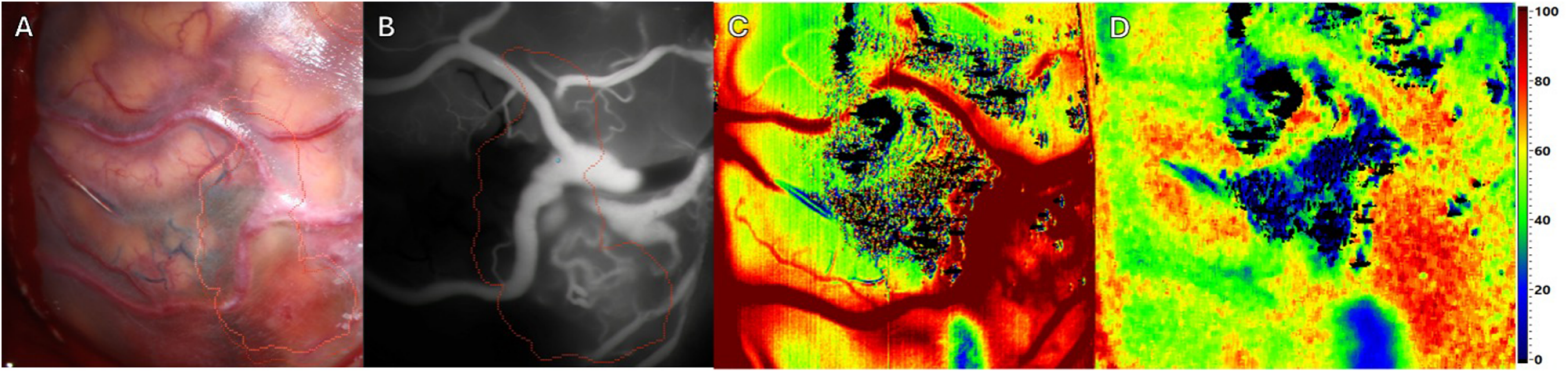
Intraoperative imaging of a superficial arteriovenous malformation (AVM) using multiple imaging modalities. Pre-resection. Four different Imaging methods in approximately equal projection in surgeons’ perspective. (**A**) Standard RGB image. The preoperative defined borders of resections are outlined using brainlab navigation (red and orange outlines). (**B**) ICG image. The previously mentioned outlines remain. (**C**) HSI image depicting tissue perfusion. (**D**) HSI image depicting tissue oxygenation.

The case was discussed preoperatively among senior neurosurgery and neuroradiology experts, and it was decided to perform a preoperative embolization attempt of two feeding branches from the MCA. Endovascular embolization attempt of two feeding branches from MCA was performed using Squid 18 (Balt, Irvine, CA, USA) (see Figure 1E). Afterwards, microsurgical augmented-reality (AR) assisted microsurgical resection (Zeiss Kinevo, Zeiss, Germany) with Surgical Hyperspectral imaging and ICG Near-infrared Evaluation (SHINE) was performed. HSI (see Figures 2C,D) was evaluated regarding oxygenation and perfusion index.

Prior to resection (see Figure 2A), multiple imaging modalities were applied. As expected ICG VA depicted a reliable perspective of the AVM with corresponding feeding and draining vessels (see Figure 2B). HSI manages to create an consistent map depicting perfusion (see Figure 2C). In addition HSI provides information regarding tissue oxygenation adjacent to the targeted vessels (see Figure 2D).

**Figure 3:**
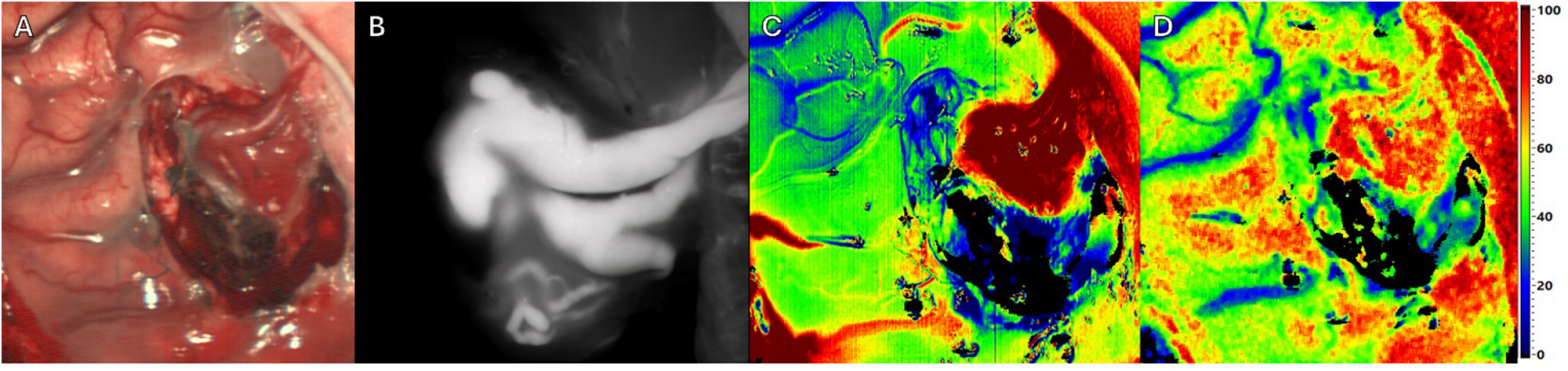
Intraoperative imaging of a superficial arteriovenous malformation (AVM) using multiple imaging modalities. Throughout resection. Four different Imaging methods in approximately equal projection in surgeons’ perspective. (**A**) Standard RGB image. (**B**) ICG image. (**C**) HSI Image depicting perfusion. (**D**) HSI Image depicting tissue oxygenation.

Throughout resection (see Figure 3A), HSI (see Figures 3C,D) continues to depict vessels whose connection to the AVM has been interrupted during surgery. Here a reduction in perfusion and oxygenation was observed, while consistent information regarding perfusion of the AVM with ICG visualization remains (see Figure 3B).

**Figure 4:**
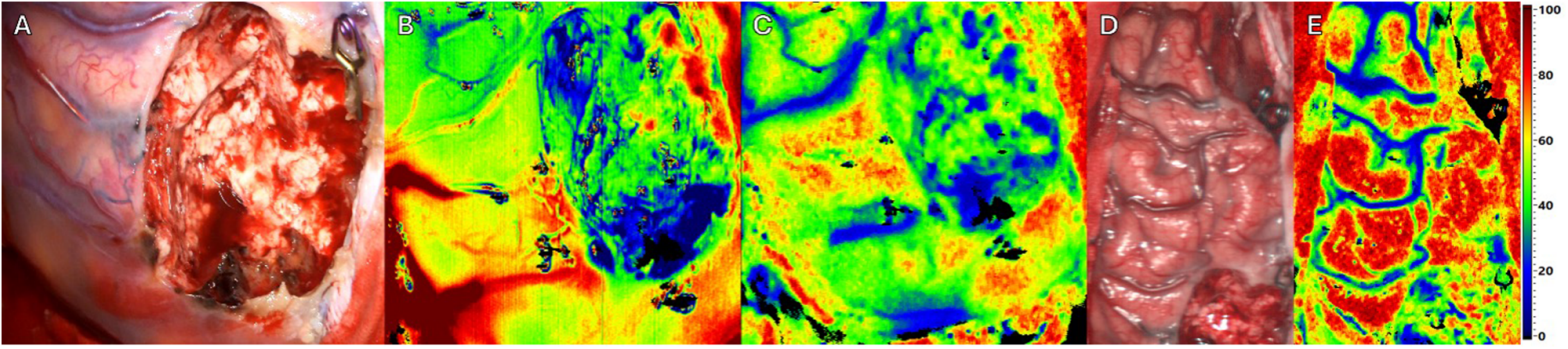
Intraoperative imaging of a superficial arteriovenous malformation (AVM) using multiple imaging modalities. Resection complete. Three different Imaging methods in approximately equal projection in surgeons’ perspective. (**A**) Standard RGB image. (**B**) HSI Image depicting perfusion. (**C**) HSI Image depicting tissue oxygenation. (**D)** Standard RGB Image of the affected hemisphere of the patients’ brain. **(E)** HSI Image of the affected hemisphere depicting tissue oxygenation.

Next to resection site (see Figures 4A,D), intraoperative HSI showed an overall increase of perfusion and oxygenation of the AVM margin (see Figures 4B,C) and ipsilateral frontal brain lobe (see Figure 4E) after complete AVM resection. Except for the resection site, no areas with significant reduction in oxygenation could be detected. The flow-related aneurysms were subsequently treated with straight aneurysm clips. Immediately postoperatively, the patient presented a novel mild aphasia, which might be the result of the normal perfusion pressure breakthrough (NPPB). HSI intraoperatively showed the increase of oxygenation and hyperemia, which might be a diagnostic indicator for this syndrome and the resulting new deficit (see Supplementary Video). The postoperative cranial CT showed no signs of acute pathology, bleeding or infarction. Postoperative DSA revealed complete excision of the AVM and occlusion of the flow-related aneurysms by two straight clips. The mild aphasia completely regressed until discharge of the patient at the sixth day after surgery.

## 3 Discussion

In this case report, we detail our initial experience using Hyperspectral Imaging (HSI) and Indocyanine Green Videoangiography (ICG VA) together to visualize perfusion during brain AVM surgery. The combination of these advanced imaging techniques enhances the intraoperative assessment of vascular structures and tissue perfusion, offering a comprehensive approach to managing complex AVMs.

ICG VA is a well-established fluorescent dye activated by near-infrared light (805 nm). Its fluorescence properties make it an invaluable tool for real-time angiography, allowing surgeons to visualize vascular anatomy and assess tissue perfusion dynamically. The utility of ICG VA in neurosurgery has been extensively documented, particularly for its role in the visualization of blood flow in small vessels and the assessment of vascular integrity during aneurysm surgery. Studies have demonstrated that ICG VA can accurately depict vascular flow and identify incomplete aneurysm occlusion or stenosis, which is crucial for ensuring optimal surgical outcomes (4, 5). In our case, the use of ICG VA allowed for the clear delineation of AVM feeding and draining vessels, facilitating a precise and safe resection. HSI represents a cutting-edge technology that captures a broad spectrum of light beyond the visible range, offering detailed spectral information about tissue properties (9). HSI does not require any contrast agents, making it a non-invasive and fast imaging modality. This technique provides several false-color images representing physiological parameters such as tissue oxygenation, tissue water content, and perfusion indices, enabling a thorough analysis of tissue viability and perfusion status (16, 17). For instance, HSI has been explored for its potential to enhance the visualization of brain tumors and assess cerebral perfusion (10, 18). Furthermore, in neurovascular field such as Moyamoya disease, HSI has been used to predict cerebral hyperperfusion syndrome post-bypass surgery, highlighting its ability to provide detailed perfusion maps and guide surgical decisions (18). In our case, HSI complemented ICG VA by offering a more comprehensive analysis of tissue perfusion and vascular integrity, thereby improving the accuracy of the AVM resection. For the present workflow we use the TIVITA® system produced by Diaspective Vision GmbH (Am Salzhaff-Pepelow, Germany). The exact technical characteristics of this imaging method have been previously published (19). This HSI system enables us to measure a spectrum of a range from 500 nm to 1,000 nm in wavelength. Besides parameters like oxygen saturation, perfusion, and content of hemoglobin, water, and fat, this contactless and contrast-agent-free imaging process shows detailed spectroscopy of several regions of interest with their absorbance spectra within a few seconds. The combination of HSI and ICG VA in this case provided a synergistic approach to intraoperative imaging. While ICG VA offered real-time visualization of blood flow and vascular structures, HSI provided detailed spectral data that allowed for the visualization of surrounding tissue characteristics (e.g., oxygenation, perfusion, water content) and identification of subtle perfusion anomalies. This dual-modality approach might enhance the precision and safety of the AVM surgery, ensuring a more complete resection with minimal risk of residual nidus. It might be worth mentioning at this point that, due to the rapid wash-out phase of ICG in the brain, the HSI images are likely not affected by artifacts. For example, in procedures involving the intestines, HSI typically needs to be performed before the administration of ICG (19, 20). However, the current workflow is limited by its ergonomics because the present HSI system is not integrated in the microscope and provides no real-time guidance in terms of hyperspectral video imaging. One of the potential future main advantages of more advanced and integrated HSI systems in the field of AVMs might be its ability to provide detailed spectral information of surrounding brain tissue before and after surgical excision of the AVM without the need for intravenous contrast agents, reducing the risk of adverse reactions and simplifying the imaging process. Additionally, HSI's fast acquisition time and non-contact nature make it a practical tool for intraoperative use in terms of potential low risk for surgical site infections and brain injury. Conversely, the real-time feedback and proven efficacy of ICG VA in visualizing vascular structures make it an indispensable tool in complex neurosurgical procedures. An important consideration and aspect in future HSI studies investigating AVM surgery is the change in perfusion of brain tissue surrounding the AVM following resection. This phenomenon, known as normal perfusion pressure breakthrough (NPPB) syndrome, can lead to hemorrhage and edema in the surrounding normal brain tissue due to the sudden restoration of normal perfusion pressures to previously hypoperfused areas (21). This occurs because the arteries supplying the AVM become dilated and lose their ability to autoregulate blood flow. Immediately following AVM removal, these arteries are unable to constrict adequately, resulting in hyperperfusion and potential damage to surrounding brain tissue. A study by Asgari et al. (22) demonstrated that intraoperative near-infrared spectroscopy (NIRS) with surface application on the cortex could detect hyperemia in the cortex adjacent to AVMs after resection. Their findings indicated significant increases in cortical oxygen saturation (StO_2_) and blood volume (BV) post-resection, suggesting that these parameters are indicative of hyperemia and potential complications. As we observed similar findings with increased saturation of oxygen (see Figure 4E) of the surrounding brain regions after AVM resection with novel postoperative neurological symptoms, our parameters suggest that HSI as a contrast-agent free and contactless quick-to-use modality could play a crucial role in identifying patients at risk of NPPB by providing detailed maps of tissue perfusion and oxygenation, allowing for real-time monitoring and identification of those patients at risk for NPPB after AVM surgery. Using HSI, we observed a continuous increase of St0_2_ in identical ipsilateral frontal brain regions next to the AVM resection site from beginning of intradural dissection to achievement of total AVM excision (see Supplementary Video). Despite the present case is the first to show HSI as an intraoperative imaging tool in AVM brain surgery and its potential use in the identification of those patients at risk for NPPB, the observed findings in HSI and the perioperative events reported is an isolated finding and needs to be evaluated using a larger set of AVM patients undergoing surgical excision. Furthermore, the present version of the institutional HSI system does not allow real-time analysis of brain perfusion at video-rate, which might provide more insight into the flow dynamics and changes of perfusion during the microsurgical steps of AVM resection. However, we introduced this dual intraoperative imaging approach (HSI & ICG VA) at the present institution and will further evaluate the use of HSI in neurovascular surgery as part of an ongoing prospective study.

## 4 Conclusion

We successfully demonstrated the first combined use of HSI and ICG VA for visualizing perfusion during brain AVM surgery. This dual-modality approach provided comprehensive intraoperative assessment, enhancing surgical precision and safety. Future integration of HSI into the surgical workflow holds potential for further improving outcomes in AVM resection by enabling detailed, contrast-agent-free perfusion mapping.

## Data Availability

The original contributions presented in the study are included in the article/Supplementary Material, further inquiries can be directed to the corresponding author.
