# Supplementary figures and images for: Surgical Hyperspectral imaging and Indocyanine green Near-infrared Examination (SHINE) for brain arteriovenous malformation resection: a case report on how to visualize perfusion
Source: Front Surg. 2024 Oct 18;11:1477920. doi: 10.3389/fsurg.2024.1477920 (PMC11527785; doi:10.3389/fsurg.2024.1477920)

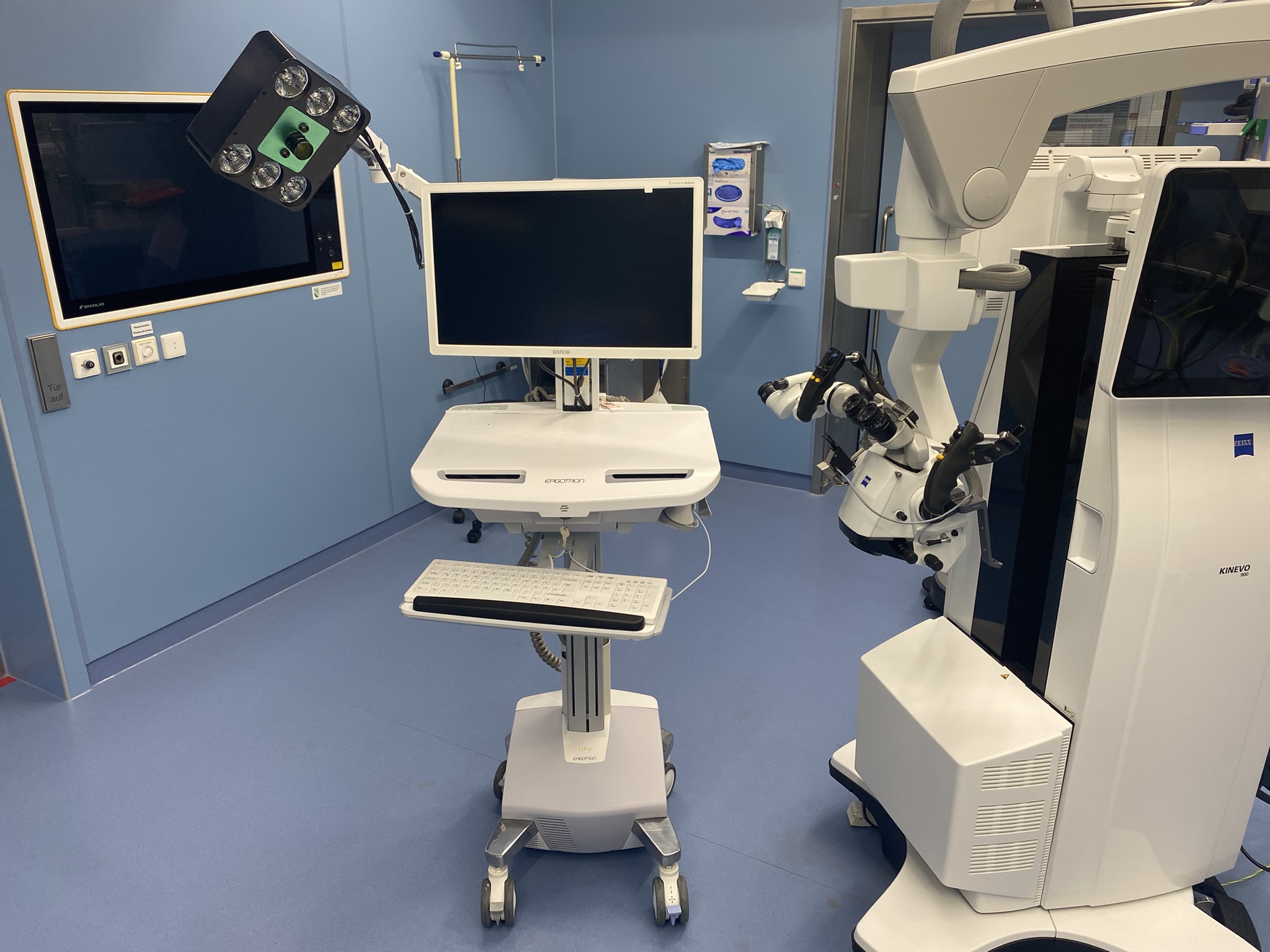

Supplement: Supplementary file 2 [file Image1.jpeg]
